# Design and Performance of 3D-Printed Hybrid Polymers Exhibiting Shape Memory and Self-Healing via Acrylate–Epoxy–Thiol–Ene Chemistry

**DOI:** 10.3390/polym17192594

**Published:** 2025-09-25

**Authors:** Ricardo Acosta Ortiz, Alan Isaac Hernández Jiménez, José de Jesús Ku Herrera, Roberto Yañez Macías, Aida Esmeralda García Valdez

**Affiliations:** Macromolecular Chemistry and Nanomaterials Department, Center for Research in Applied, Chemistry, Blvd. Enrique Reyna #140, Saltillo 25297, Mexico; alan.hernandez.d21@ciqa.edu.mx (A.I.H.J.); jesus.ku@ciqa.edu.mx (J.d.J.K.H.); roberto.yanez@ciqa.edu.mx (R.Y.M.); aida.garcia@ciqa.edu.mx (A.E.G.V.)

**Keywords:** 3D printing, acrylate, thiol-ene, anionic ring opening polymerization, epoxide, photopolymerization, shape memory, self-healing

## Abstract

This study presents a novel strategy for designing photocurable resins tailored for the additive manufacturing of smart thermoset materials. A quaternary formulation was developed by integrating bis(2-methacryloyl)oxyethyl disulfide (DADS) with an epoxy/thiol-ene system (ETES) composed of diglycidyl ether of bisphenol A (EP), pentaerythritol tetrakis(3-mercaptopropionate) (PTMP), and 4,4′-methylenebis(N,N-diallylaniline) (ACA4). This unique combination enables the simultaneous activation of four polymerization mechanisms: radical photopolymerization, thiol-ene coupling, thiol-Michael addition, and anionic ring-opening, within a single resin matrix. A key innovation lies in the exothermic nature of DADS photopolymerization, which initiates and sustains ETES curing at room temperature, enabling 3D printing without thermal assistance. This represents a significant advancement over conventional systems that require elevated temperatures or post-curing steps. The resulting hybrid poly(acrylate–co-ether–co-thioether) network exhibits enhanced mechanical integrity, shape memory behavior, and intrinsic self-healing capabilities. Dynamic Mechanical Analysis revealed a shape fixity and recovery of 93%, while self-healing tests demonstrated a 94% recovery of viscoelastic properties, as evidenced by near-overlapping storage modulus curves compared to a reference sample. This integrated approach broadens the design space for multifunctional photopolymers and establishes a versatile platform for advanced applications in soft robotics, biomedical devices, and sustainable manufacturing.

## 1. Introduction

Smart polymers are advanced materials capable of responding to external stimuli such as temperature, light, pH, or electric fields, which induce changes in their physical properties including shape, stiffness, or color. This versatility makes them highly valuable for various applications [1,2]. Examples include shape memory polymers (SMPs), which revert to their original shape after deformation [3], and self-healing polymers (SHPs), which can autonomously repair themselves following damage [4]. Their ability to adapt to environmental changes makes smart polymers suitable in fields like medicine, aerospace, electronics, and beyond [5].

The integration of smart materials into 3D printing technology significantly enhances the capabilities and applications of additive manufacturing [6,7]. These materials provide adaptive, functional, and sustainable solutions, addressing the evolving requirements of diverse sectors, including healthcare and consumer goods. The convergence of SMP with additive manufacturing offers a powerful platform for fabricating intelligent, reconfigurable structures with unprecedented design freedom and functionality [8]. This synergy introduces novel functional capabilities and broadens the applications of SMPs. Due to their automation-friendly nature and cost-effectiveness, 3D printed SMPs have applications in numerous areas, such as smart robotics, adaptive antennas, medical drug delivery systems, and regenerative tissue engineering [9]. SMPs function typically through an interaction between shape-fixing and shape-switching components.

Conversely, SHP represents an innovative class of materials within the 3D printing domain, offering benefits that significantly enhance the performance, durability, and sustainability of printed parts [10]. Traditional 3D printed components often suffer from microcracks and damage leading to potential catastrophic failure or necessitating complete part replacements. SHP incorporate dynamic bonds, either through reversible covalent chemistry (such as disulfide exchange) or supramolecular interactions, enabling autonomous repair of such damages [11]. This self-repair mechanism extends the operational life of parts by restoring mechanical integrity and minimizing the probabilities of failure during service. Components made from these polymers require less frequent maintenance due to their ability to self-heal minor damage. In scenarios where downtime is costly or part replacement is impractical, self-healing could reduce maintenance expenses and logistical challenges. Additionally, the reduced need for replacements minimizes resource consumption and waste, which is particularly beneficial in industries employing 3D printing for prototypes and low-volume production runs [12].

Hybrid photopolymer formulations, combining multiple reactive chemistries in a single “ink,” enhance material performance for vat-based 3D printing. The polymers derived from these systems offer distinct properties, such as tailored mechanical characteristics, reduced brittleness, modulated glass-transition temperatures (T_g_), improved chemical resistance, controlled polymerization shrinkage, gradient materials, sequential curing strategies, and the integration of customized electrical, thermal, or biological properties along with stimuli-responsive functions. Several studies have documented the development of hybrid inks for 3D printing, resulting in photopolymers with enhanced properties. For instance, Huang et al. [13] introduced a hybrid photosensitive resin for 3D printing, combining bisphenol A–type epoxy diacrylate, polyfunctional acrylates, and a cycloaliphatic diepoxide. This resin uses both free radical and cationic polymerization under UV light, achieving high dimensional accuracy with minimal shrinkage. Latean et al. [14] developed hybrid acrylic/epoxy materials for DLP 3D printing. The formulation blends hexanediol diacrylate and cycloaliphatic epoxy resin, with glycidyl methacrylate as a coupling agent. This hybrid shows rapid curing, impact toughness, and thermal stability. Casado et al. [15] explored 3D printable hybrid acrylate-epoxy dynamic networks. These materials underwent UV-induced polymerization followed by thermal cross-linking, enabling post-curing repair and recycling through dynamic exchange reactions. Zhao et al. [16] compared silicone-epoxy/acrylate hybrid polymers using pure photopolymerization and dual-curing mechanisms. The dual-curing method, involving photo and thermal curing, produced objects with enhanced mechanical and thermal properties. These studies emphasize the benefits of hybrid resins in 3D printing, such as improved mechanical properties, reduced shrinkage, and reprocessability. Post-curing treatments and initiator system optimization are crucial for enhancing curing speed and final part performance.

This study presents a novel approach to smart photopolymer design by integrating four distinct polymerization mechanisms—radical photopolymerization, thiol-ene photopolymerization, thiol-Michael addition, and anionic ring-opening polymerization—into a single, synergistic resin formulation. Unlike previously reported hybrid systems that typically combine only two reactive chemistries and often rely on thermal post-curing, our quaternary formulation enables the simultaneous formation of polyacrylate, polythioether, and polyether networks under ambient conditions. The exothermic nature of DADS photopolymerization initiates and sustains the curing of the ETES components without external heating, allowing efficient 3D printing of thermoset materials with advanced functionalities. This integrated strategy not only enhances mechanical integrity and shape memory performance but also introduces intrinsic self-healing behavior through dynamic disulfide exchange. To realize this multifunctional platform, a hybrid quaternary formulation was developed, consisting of an acrylate monomer bearing disulfide bonds, an aromatic epoxy resin, a tetraallyl ditertiary amine curing agent, and a tetrafunctional thiol. The formulation’s chemical reactivity was monitored using Real-Time Infrared (RT-IR) spectroscopy, while its exothermic behavior during photopolymerization was quantified via optical pyrometry (OP). Shape memory performance was evaluated through Dynamic Mechanical Analysis (DMA), and self-healing capability was assessed by thermally rejoining bisected specimens. To investigate interfacial enhancement, two coupling agents were incorporated into the formulation, and their effects were analyzed via DMA and compared to the baseline polymer lacking these additives. Tensile testing was conducted to determine key mechanical properties, including elastic modulus, tensile strength, toughness, and elongation at break.

## 2. Materials and Methods

### 2.1. Reagents

**Scheme 1:**
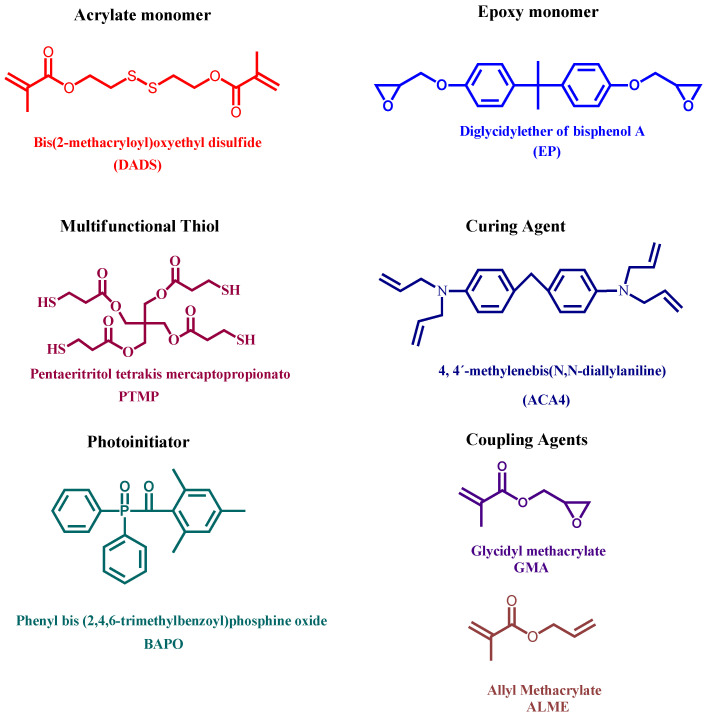
Chemical structure of chemicals used in this study.

Bis(2-methacryloyl)oxyethyl disulfide, 90% purity (DADS), diglycidyl ether of bisphenol A, technical grade (EP), pentaerythritol tetrakis (3-mercaptopropionate) 95% purity, (PTMP), dimethoxyphenyl acetophenone 99% purity, (DMPA), Phenyl bis (2,4,6-trimethylbenzoyl)phosphine oxide 97% purity, (BAPO), allyl methacrylate 98% purity (ALME), glycidyl methacrylate 97% purity (GMA), trimethylolpropane triacrylate 90% purity, (ACR1), were all purchased from Merck in Toluca Mexico and used as received. The curing agent 4,4′-methylenebis (N, N-diallylaniline) (ACA4) was synthesized according to a previous reported method [17]. The chemical structures of these compounds are depicted in Scheme 1.

### 2.2. Preparation of the Quaternary Formulation

To prepare the quaternary formulation, a 1:1 equivalent ratio was established between the acrylate monomer DADS and an epoxy/thiol-ene system (ETES). The ETES component comprises one equivalent of the epoxy resin EP and a 40 mol% proportion of the thiol-ene system (TES). The TES itself is a stoichiometric mixture of the curing agent ACA4 and the multifunctional thiol PTMP, both of which are tetrafunctional and thus contribute to a highly crosslinked network. To initiate photopolymerization, the photoinitiator BAPO was incorporated into the formulation at 1 mol% with respect to PTMP. Table 1 details the gram-scale quantities of each component in the formulation.

### 2.3. Real-Time Infrared Spectroscopy (RT-FTIR)

The RT-FTIR analysis was conducted using a Thermofisher Nicolet 6700 spectrometer (Franklin, MA, USA) and a spot-cure UV lamp Dymax Bluewave 200 (Dymax, Hartford, CT, USA), to photocure the samples. The conversion of double bonds, thiols, and epoxy groups in the hybrid formulations during photopolymerization was measured using the Series application in the OMNIC 8.0 software. The details of the setup of the equipment to determine the kinetics of photopolymerization have been previously reported [18].

### 2.4. Shape Memory Analysis by DMA

Samples for this analysis were prepared using LCD printing and assessed via a DMA Q800 (TA Instruments, New Castle, DE, USA) to evaluate shape-memory properties [19]. Using cyclic stress control mode and film tension clamps, a 0.01 N force was initially applied and balanced for 10 min. The sample was preheated to 120 °C, without data collection. Samples were then deformed with a force ramp of 0.50 N/min to 1.50 N/min, cooled at 5 °C/min to 40 °C, and held for 60 min. After cooling, the force was reduced from 0.50 N/min to 0.00010 N/min, followed by 30 min of balancing. Reheating to 120 °C at 5 °C/min and maintaining for 60 min completed one cycle. This process was repeated for four cycles. The parameters obtained by this method were the shape fixity (R_f_) and the shape recovery (R_r_) and were calculated using the following equations [20]:

R_f_ = (ε_u_ (N) − ε_p_ (N − 1))/(ε_m_ − ε_p_ (N − 1)) (1)

R_r_ = (ε_u_ (N) − ε_p_ (N))/(ε_u_ (N) − ε_p_ (N − 1)) (2)

where ε_m_ is the maximum strain under loading, ε_u_ (N) is the unloading strain of the cycle number N, and ε_p_ (N) and ε_p_ (N − 1) were residual strain in the cycle number N and N − 1.

Shape fixity (Equation (1)) refers to the polymer’s ability to retain a temporary shape after deformation and cooling. It quantifies how well the material “locks in” the deformed configuration when external forces are removed. This is typically achieved by cooling the polymer below its transition temperature (e.g., glass transition temperature, T_g_) while it is held in the desired shape. The shape-recovery ratio (Equation (2)) describes the polymer’s ability to return to its original (permanent) shape when exposed to a stimulus, such as heat, light, or moisture. This process is triggered when the polymer is reheated above its transition temperature, allowing molecular mobility and driving the reconfiguration back to its original shape.

### 2.5. Optical Pyrometry (OP)

In this study, we sought to quantify the temperature rise associated with the exothermic nature of photopolymerization reactions. We utilized optical pyrometry (OP) using a method previously reported by Crivello et al. [21], employing an industrial infrared thermometer (Omega OS550, Omega Engineering^®^, Inc., Stamford, CT, USA) with a sensitivity of ±1 °C. Formulations were prepared following the compositions detailed in Table 1. A 0.05 g sample was then placed between two 2 cm × 2 cm layers of corona-treated polypropylene (PP) film. This sample assembly was positioned horizontally on a lab jack inside an obscure chamber. The optical pyrometer was aligned vertically, directly facing the sample, while the tip of the wand of the UV lamp (Dymax Bluewave 200) was angled at 45° relative to the sample surface. The lamp intensity was set at 40 mW/cm^2^, using unfiltered radiation in the 200–800 nm range. UV irradiation of the sample and temperature measurement via optical pyrometry were initiated simultaneously, resulting in temperature versus irradiation time profiles.

### 2.6. Self-Healing Properties Determination

The cured test specimens were bisected using a razor blade and separated to demonstrate the fracture of the materials. Each specimen was then positioned within a metal mold to ensure alignment for the healing process by bringing the two halves of the specimen closer together. The assembly underwent thermal treatment on a heating plate set to 120 °C, with the temperature monitored using a laser thermometer for consistency. Photographic documentation was performed at zero time, followed by periodic visual inspections to observe the progression of the self-healing process. Successful healing was noted when the halves of the specimen fused into one piece, maintaining structural integrity independently.

### 2.7. Three-Dimensional Printing of the Quaternary Formulation

The quaternary system was printed using a Phrozen Sonic Mini 4K UV LCD printer, equipped with a 6.1-inch monochrome display and a build volume of 5.2 × 2.9 × 5.1 cm (length × width × height). The specific printing parameters employed for this process are detailed in Table 2.

### 2.8. Determination of Tensile Mechanical Properties

The tensile behavior the polymers derived from the formulations DADS/ETES 1:1 and ACR1/ETES 1:1 was assessed following the ASTM D638-22 standard. [22]. V specimens were tested under uniaxial tension using a Shimadzu AGX universal testing machine (Shimadzu Corporation, Kyoto, Japan) equipped with a 5 kN load cell, operating at a crosshead displacement rate of 2 mm/min. The elastic modulus (E) was calculated from the slope of the initial linear portion of the stress–strain (σ − ε) curve, while tensile toughness was determined by integrating the area under the curve up to the point of fracture. For each formulation, five replicate specimens were analyzed to obtain mean values and standard deviations for E, ultimate tensile strength (σ_max_), toughness, and elongation at break (ε_break_).

## 3. Results

This study explored the shape memory and self-healing properties of a polymer derived from a photocurable quaternary hybrid acrylate/epoxy/thiol-ene formulation. Shape memory properties of this polymer were due to rigid (polyethers and polyacrylates) and soft segments (polythioethers) in the crosslinked co-network, while self-healing occurred through disulfide-thiolate exchanges. Our group previously reported that quaternary formulations with multifunctional acrylates and an epoxy/thiol-ene system (ETES) can be 3D printed at room temperature, despite ETES normally requiring photopolymerization at 85 °C [23,24]. Multifunctional acrylates chosen had highly exothermic photopolymerization, reaching up to 200 °C, which facilitated concurrent photopolymerization of ETES at room temperature. The ETES included an aromatic epoxy resin, an aromatic tetraallyl ditertiary amine curing agent, a tetrafunctional thiol, and a photoinitiator.

**Scheme 2:**
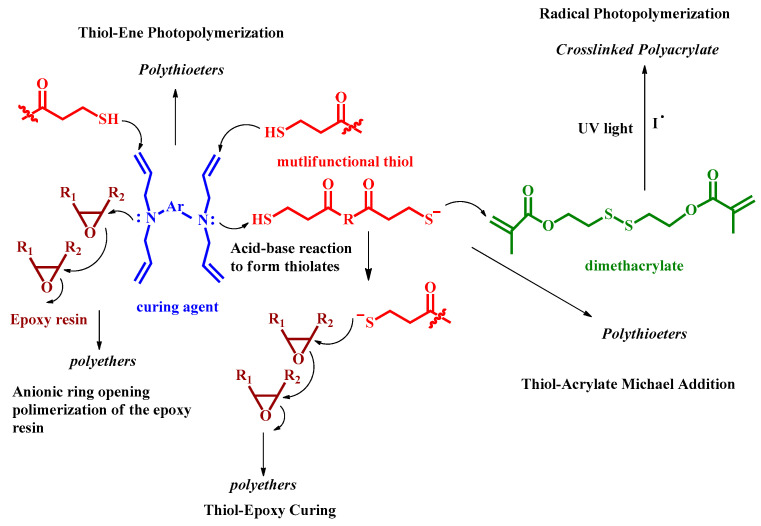
Proposed Mechanism of the photopolymerization of the quaternary photocurable formulation DADS/ETES. Adapted from [17], with permission from John Wiley and Sons, 2024.

In this research, smart polymers were prepared using principles from our previous research [17]. We used an acrylate with disulfide bonds (DADS) and an ETES. The photopolymerization involved multiple reactions: dimethacrylate photopolymerization, radical thiol-acrylate and thiol-ene photopolymerizations, thiol-acrylate Michael addition, thiol-epoxy curing, and anionic ring-opening polymerization (AROP) of epoxy groups initiated by tertiary amine groups [17]. The proposed mechanism is shown in Scheme 2.

### 3.1. Kinetics of Photopolymerization

**Figure 1:**
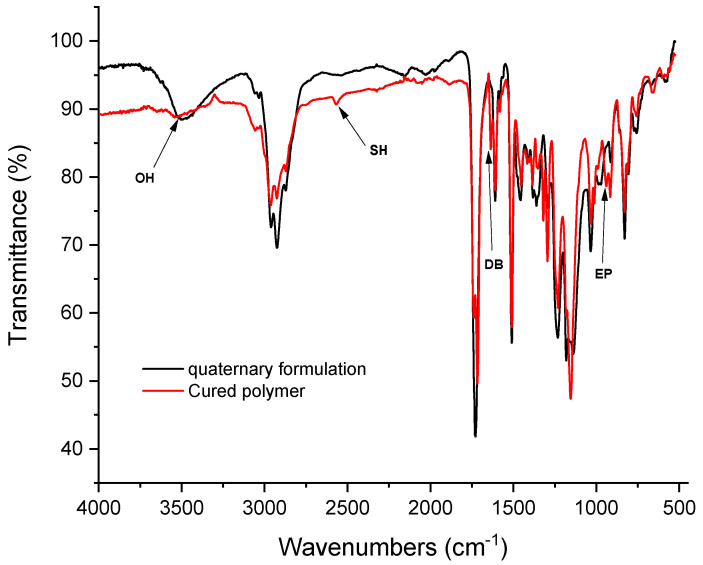
FTIR spectra of the quaternary formulation before photocuring (red curve) and after curing (black curve). The FTIR of the formulation was conducted using a KBr disk, while the cured specimen was analyzed using ATR with a diamond crystal. (OH represents hydroxyl groups, SH indicates thiol groups, DB double bonds and EP stands for epoxy groups).

The reactivity of the formulation for 3D printing, at room temperature was assessed using RT-FTIR to track photopolymerization kinetics. Figure 1 displays the comparison of the FTIR spectra of the formulation before and after photocuring, highlighting key bands: C-H stretching at 2980 and 2893 cm^−1^, thiol groups stretching at 2568 cm^−1^, C=O stretching at 1737 and 1717 cm^−1^, C=C stretching at 1643 cm^−1^ and the stretching band due to epoxy groups, at 974 cm^−1^.

The band at 1643 cm^−1^ represents overlapping acrylate and allylic C=C stretches, and the band at 1607 cm^−1^ corresponds to aromatic hydrogens. The epoxy group’s absorption was determined using the band at 974 cm^−1^. Post-curing, the absorbance of the double bonds and thiol groups decreased almost quantitatively, and the epoxy band decreased by 60%. It is also evident the appearance of a wide band centered at 3400 cm^−1^ due to the hydroxyl groups formed during the AROP of the epoxy resin.

**Figure 2:**
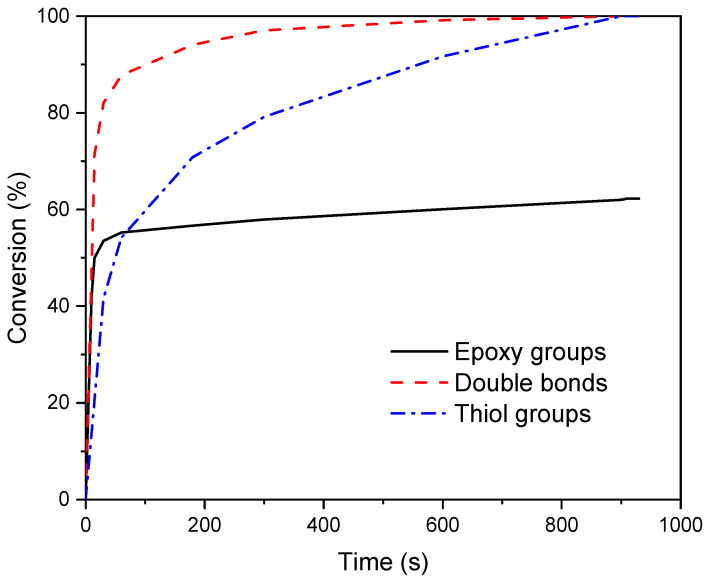
RT-FTIR kinetics of photopolymerization of the quaternary formulation including DADS, EP, ACA4 and PTMP in the presence of BAPO at 1 mol% using UV-Vis radiation of 40 mW/cm^2^.

Figure 2 presents real-time FTIR monitoring of the photopolymerization kinetics in the quaternary system composed of DADS, EP, PTMP, ACA4, in a 1:1:0.4:0.4 molar ratio, respectively. Due to spectral overlap, the curve for double bond conversion reflects the combined kinetics of methacrylate and allylic moieties. The steep initial slope indicates a rapid reaction involving acrylate homopolymerization, thiol-acrylate and thiol–ene radical photopolymerizations between thiols and dimethacrylate and allyl groups of ACA4. The reactivity of the double bonds stood out, achieving 82% conversion within the first 30 s of irradiation and reaching a final conversion of 99% after 1000 s. This results in the fast formation of a polyacrylate–polythioether network. In contrast, the epoxide conversion curve shows a biphasic profile: a sharp initial rise, attaining a conversion of 53% within 30 s, driven by the basic nature of tertiary amines, thiolates, and slightly basic polythioethers derived from the thiol-ene photopolymerization, followed by a slower phase as unreacted epoxides become sterically trapped within the rigidifying matrix, reaching 62% conversion. The exothermic nature of dimethacrylate polymerization aids in network relaxation and sustains AROP of epoxide groups and thiol–epoxy reactions even after vitrification.

The thiol conversion profiles exhibit complex behavior reflective of the network’s multifunctional reactivity. Initially, thiol groups participate in thiol–ene photopolymerization, concurrently with acid–base interactions between thiols and tertiary amines present in the curing agent, generating thiolate species, which are highly nucleophilic and initiate further reactions. These thiolates engage in thiol–Michael addition with acrylate moieties, forming polythioether segments that contribute to network flexibility and uniformity. Additionally, thiolates undergo thiol–epoxy ring-opening reactions, promoting anionic polymerization of the epoxy resin and enhancing crosslink density. Beyond primary network formation, thiolates also interact with disulfide bonds embedded in the polyacrylate matrix, facilitating dynamic thiolate–disulfide exchange. This reversible mechanism under thermal stimuli enables topological rearrangement and imparts self-healing capabilities to the material. Moreover, thiyl radicals formed during the thiol-ene photopolymerization can also dimerize into disulfide species, which could also participate actively in thiolate–disulfide exchange.

This kinetic profile underscores the complex, multi-pathway reactivity of the system, with each group contributing to the formation of a crosslinked co-network of poly(acrylate-co-ether-co-thioether) type. This crosslinked network display shape memory and self-healing properties.

### 3.2. Determination of Exothermicity of Hybrid Photopolymerization

**Figure 3:**
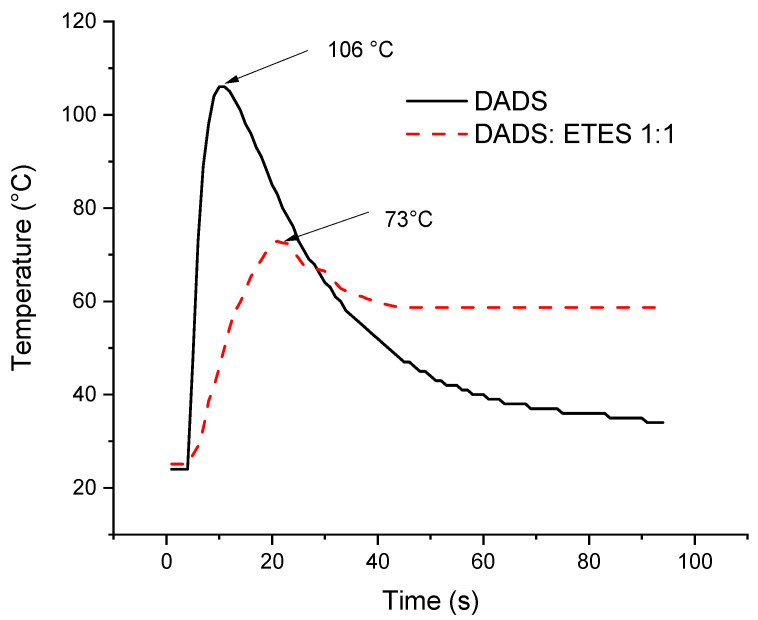
Temperature profiles during the photopolymerization of pristine DADS and the DADS–ETES (1:1) formulation.

To confirm the exothermic nature of the quaternary formulation and to assess whether the heat release could serve as a driving force for the ETES reaction, OP was employed. Figure 3 compares the thermal evolution of two systems: the photopolymerization of pristine DADS and the quaternary system containing a 1:1 ratio of DADS:SETE. In the first case, a rapid and intense exothermic response was observed, characterized by a steep temperature rise that peaked at 106 °C within just 15 s. This sharp thermal surge indicates a fast-crosslinking reaction with a high enthalpic release, which may lead to premature gelation and limited working time. In contrast, the DADS:SETE 1:1 formulation demonstrated a more controlled and gradual temperature increase, reaching a maximum of 73 °C at approximately 25 s. More importantly, this system maintained a thermal plateau around 60 °C for over 100 s, suggesting that the formulation possesses sustained reactivity and can provide a stable thermal environment that helps the endothermic ETES photopolymerization to proceed. Additionally, the moderated exotherm mitigates thermal stress and shrinkage effects, enhancing dimensional fidelity and mechanical integrity. This behavior is particularly advantageous for room-temperature 3D printing. The extended thermal window allows for better flow, shape retention, and potential fusion between deposited layers before gelation.

### 3.3. The Use of Coupling Agents GMA and ALME in the Hybrid Formulations

**Scheme 3:**
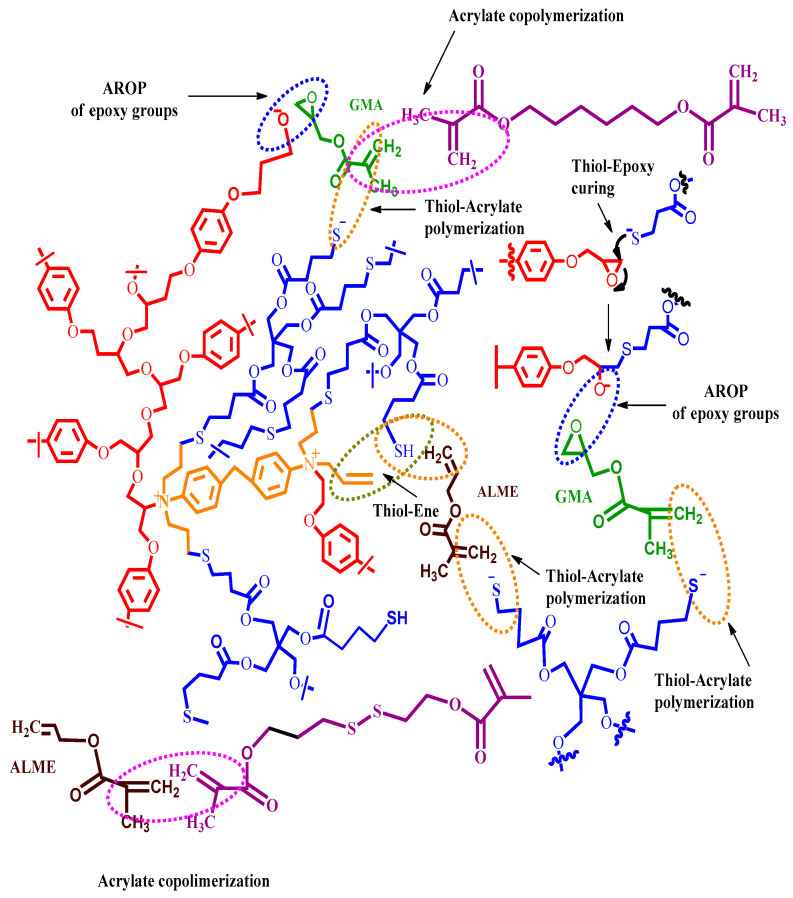
Proposed mode of action of the coupling agents GMA and ALME in the quaternary formulation with DADS/EP/ACA4/PEMP 1:1:0.4:0.4 when irradiated with UV at room temperature.

Considering that several types of polymerization occur during the curing process, it was decided to test two coupling agents, namely, glycidyl methacrylate (GMA) and allyl methacrylate (ALME). In the multifunctional photopolymerizable system composed of DADS, EP, PTMP, and the curing agent ACA4, the strategic incorporation of GMA or ALME as coupling agents plays a role in enhancing network homogeneity and interconnectivity. GMA possesses two orthogonal reactive functionalities: a methacrylate moiety capable of radical copolymerization with DADS, and an epoxide group that can also copolymerize with EP or undergo AROP by the action of thiolates or tertiary amine groups. ALME also features bifunctionality: an allyl group and a methacrylate group, each reactive under different polymerization regimes: The methacrylate moiety participates in traditional radical photopolymerization with DADS while the allyl group is reactive toward thiol–ene photopolymerization with PTMP, enabling ALME to link into the flexible polythioether subnetwork. This dual reactivity allows ALME to function as a modular crosslinker, integrating both stiff (poliacrylate-rich) and soft (polythioether-rich) domains. Its small molecular size offers flexibility in modulating network architecture, improving segmental mobility where needed, and enabling better energy dissipation or recovery behavior depending on the formulation balance. The proposed mode of action of the two coupling agents is described in Scheme 3.

**Figure 4:**
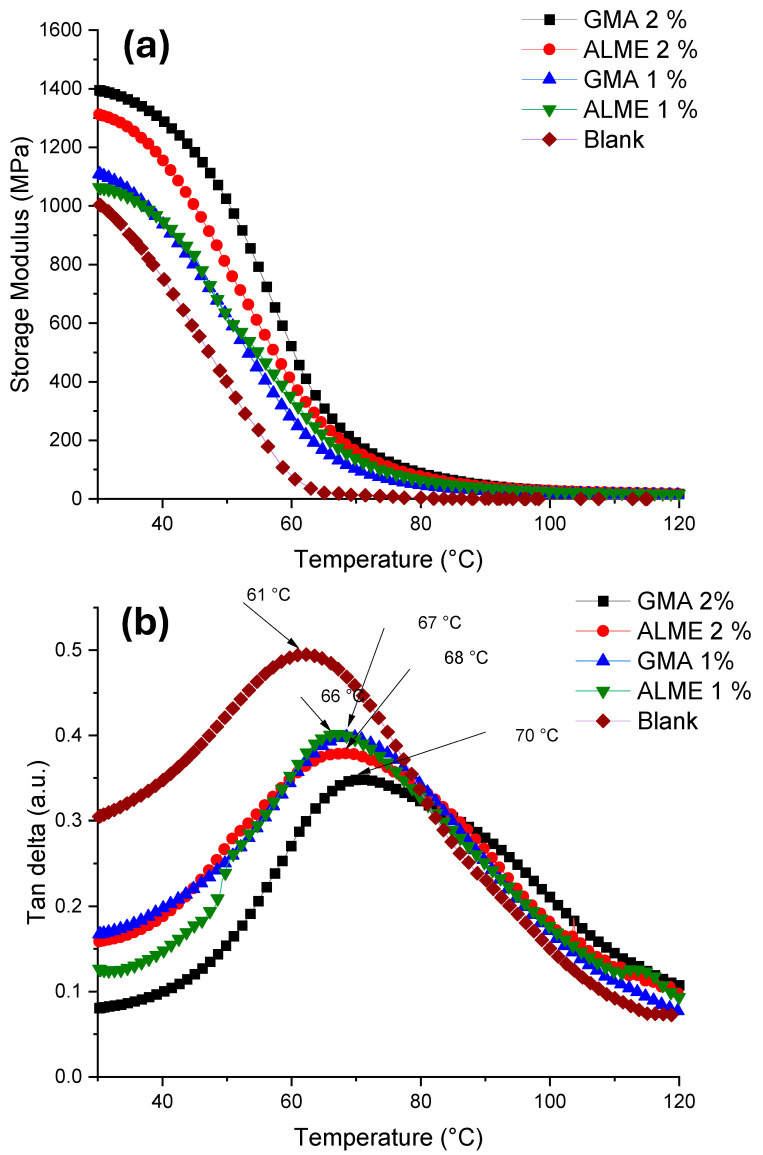
Comparison of the Storage Moduli and tan delta curves of the crosslinked polymers derived from formulation DADS/ETES 1:1. (**a**) Storage Moduli of the samples; (**b**) Tan delta curves for the samples.

Figure 4 presents the dynamic mechanical analysis (DMA) results of hybrid photopolymers formulated with DADS, EP, ACA4, and PTMP at a 1:1:0.4:0.4 equivalent ratio, modified by the addition of GMA and ALME at 1 and 2 mol%. The data reveals a clear concentration-dependent effect of both coupling agents on the viscoelastic properties of the material.

Specifically, GMA at 2 mol% exhibited the highest storage modulus (1395 MPa) across the entire temperature range, indicating superior stiffness and thermal resistance, while ALME at 2 mol% also significantly improved modulus relative to the blank, albeit to a slightly lesser extent (1312 MPa). At 1 mol%, both GMA and ALME showed moderate enhancements (1109 and 1063 MPa, respectively), confirming that increasing the concentration of the coupling agent amplifies its reinforcing effect. Additionally, both agents contributed to elevated glass transition temperatures (T_g_), with the 2 mol% formulations (70 and 68 °C) displaying tighter network formation and reduced segmental mobility compared to their 1 mol% counterparts (67 and 66 °C). The blank formulation showed the lowest modulus (1003 MPa) and T_g_ (61 °C), underscoring the critical role of coupling agents in network enhancement. Overall, the concentration of GMA and ALME helps adjust the viscoelastic properties of the quaternary smart photopolymer, confirming their value as effective coupling agents in 3D-printable materials. Their presence promotes a more uniform and connected polymer network, reducing isolated regions and improving the balance between rigid and responsive segments. This leads to better mechanical and thermal performance by enhancing structural consistency and interactions across the material.

### 3.4. Shape Memory Properties Determination

**Figure 5:**
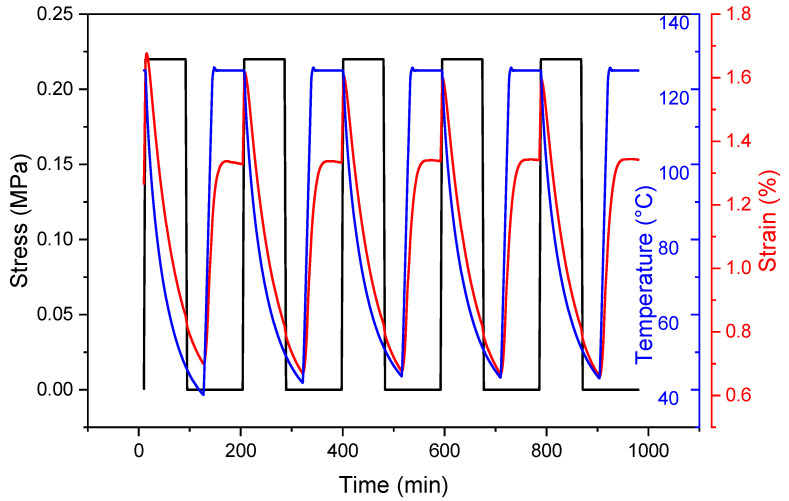
Shape memory performance determined by DMA of the poly(acrylate-co-ether-co-thioether) interconnected hybrid co-network.

Given that the obtained hybrid photopolymer includes interconnected polyacrylate, polyether and polythioether segments, a complex behavior was expected for this material. For instance, both polyacrylate and polyethers are crosslinked rigid materials while the polythioethers display certain mobility due to the flexibility of the sulfide groups. Therefore, the correct proportion of these three species is required for the polymer to display shape memory properties. Additionally, the polyethers derived from the epoxy resin contain aromatic rings that impart rigidity to the final polymer, while the polyacrylate derived from DADS monomer contain the covalent adaptive network (CAN) resulting from the disulfide bond. DMA of the polymer was performed to determine parameters like shape fixity (the ability to maintain the deformed shape) and shape recovery (the percentage of the original shape regained). Repeated cycling can reveal how the SMP’s shape memory properties change over time. The cycles can also help to understand how the SMP’s shape memory properties are affected by fatigue and/or degradation, over time. Figure 5 depicts the shape memory behavior of the prepared polymer. Under thermal cycling, the material demonstrated repeatable and well-defined shape memory effects. During heating, strain increases, and stress drops as the network transitions through its T_g_. On cooling, rigid domains vitrify, restoring internal tension and decreasing strain, effectively fixing the temporary shape. The material’s behavior underscores its efficient energy storage and release capabilities, with an average shape fixity of 93% and shape recovery of 93% in each of the four cycles, according to Table 3. This system demonstrates robust and reliable shape memory performance. A recurring strain plateau near 1.35%, appears during heating, indicative of a soft-segment response where deformation occurs at near-constant strain.

This plateau confirms that the material is undergoing maximum segmental mobility and energy dissipation at that point, precisely where soft domains activate, while rigid domains begin to yield. The fact that this stress plateau occurs reproducibly in every cycle, with nearly identical stress and strain values, strongly supports the hypothesis that it is driven by intrinsic, architecture-governed viscoelastic transitions and not to degradation or fatigue. Following the plateau, with increasing temperature and applied force, the strain reached a maximum of 1.6%.

The interplay of multiple crosslinking chemistries generates a hybrid network capable of storing elastic energy and releasing it efficiently upon heating. For instance, the rigid domains are responsible for stress accumulation and shape fixing. The polyacrylates, formed via radical photopolymerization of DADS, establish permanent crosslinked segments that remain dimensionally stable below T_g_, while polyethers, produced by base-catalyzed ring opening of bisphenol A diglycidyl ether, introduce aromatic-rich, high-T_g_ segments. These are structurally stiff and contribute to stress buildup during cooling and strain fixation in the low-temperature regime. These rigid domains anchor the material’s shape and resist deformation at lower temperatures, explaining the increased stress and stability of strain during cooling. The flexible switching segments such as the polythioethers generated via thiol–ene photopolymerization, introduce flexible chains. As temperature rises, these segments mobilize, allowing for elastic strain recovery. These flexible domains respond to heat by softening early, enabling large strain at low stress, which explains the gradual increase in strain after the plateau.

**Figure 6:**
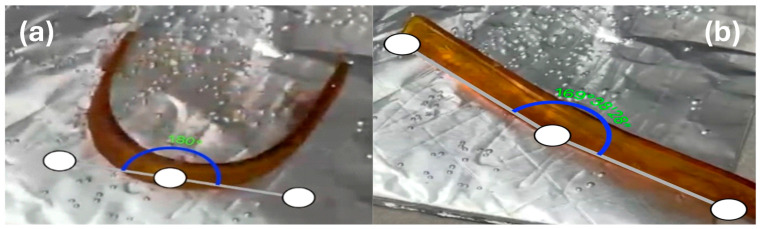
Shape memory demonstration: (**a**) specimen in its temporarily deformed U-shape, (white reference markers were placed at the ends and midpoint of the specimen to monitor deformation during bending); (**b**) post-recovery after thermal stimulus, showing relaxed curvature (169°), (white reference markers were placed at key positions along the specimen to facilitate angle measurement during deformation).

An additional shape memory evaluation was conducted via thermomechanical programming, involving deformation above T_g_, shape fixation, and thermal recovery using pre-programmed U-shaped specimens derived from the quaternary formulation (see Figure 6). These samples were initially deformed at temperatures above their glass transition temperature (T_g_), allowing temporary shape fixation. Subsequently, each specimen was immersed in a thermal stimulus bath maintained 10 °C above its T_g_, triggering shape recovery. Remarkably, the polymer demonstrated rapid actuation behavior, reverting toward its original configuration in just 6 s. The final recovered shape exhibited an angle of 169°, approaching the pre-deformation angle of 180°, which corresponds to a 93% recovery efficiency, a result consistent with the previous DMA data. This rapid and substantial recovery confirms that the polymer network possesses highly effective shape memory characteristics, enabled by the synergistic contribution of covalent crosslinks and flexible segments.

### 3.5. Self-Healing Process of the 3D Printed Test Specimens

**Figure 7:**
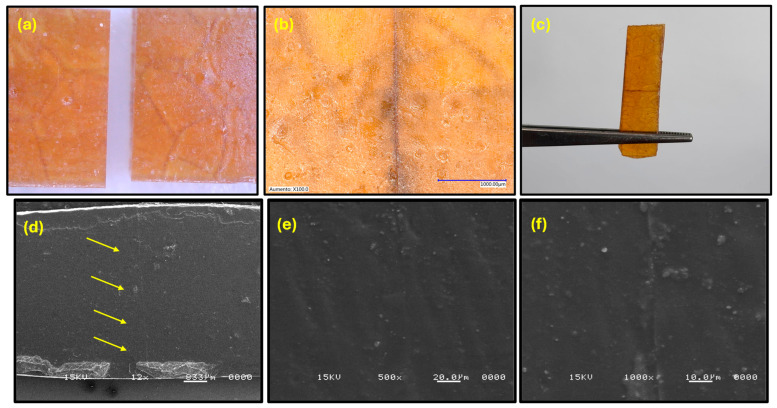
Summary of the healing process of test specimens: (**a**) bisected specimens derived from quaternary formulations; (**b**) healed specimen after 12 h at 120 °C; (**c**) self-standing specimen following thermal healing treatment; (**d**) SEM micrograph of the healed interface at 12× magnification (yellow arrows indicate the interface between the joined pieces); (**e**) SEM micrograph of the healed interface at 500×; (**f**) SEM micrograph of the healed interface at 1000×.

A study to determine the self-healing properties of the hybrid photopolymer was undertaken. The series of photographs and micrographs in Figure 7 illustrates the thermally triggered self-healing behavior in the polymer network derived from the quaternary formulation involving acrylate, epoxy, and thiol–ene chemistries. In Figure 7a, the sample is visibly split into two clean fragments, simulating mechanical damage. Figure 7b shows a seamless rejoining of the fracture plane, with no visible gap or misalignment, after thermal treatment at 120 °C for 12 h. Finally, Figure 7c demonstrates the mechanical integrity restored post-healing, as the sample supports its own weight without external reinforcement which is a strong indicator of bulk-level reformation of the network across the fractured interface. Figure 7d–f present SEM micrographs of the healed interface at increasing magnifications, illustrating the effectiveness of the healing process and the integrity of the rejoined surfaces.

**Scheme 4:**
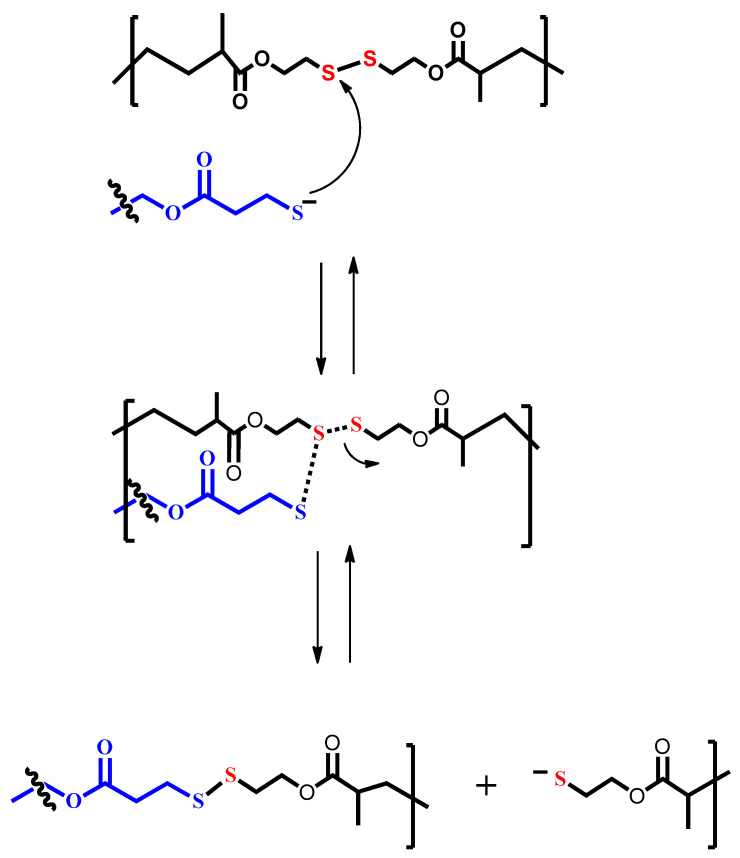
Mechanism of self-healing by the reversible disulfide bonds in the presence of thiolate species.

**Figure 8:**
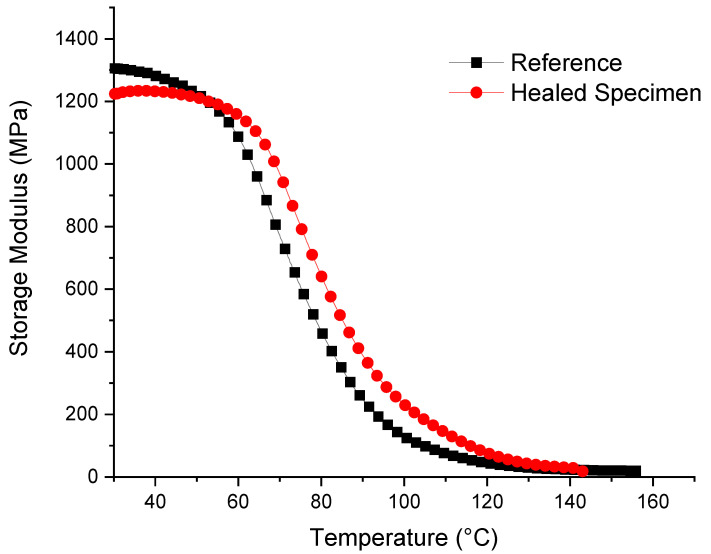
Comparison of the Storage Modulus vs. Temperature curves for a reference test specimen that was not cut, and of a healed specimen that was bisected and then reformed by means of thermal treatment for 20 h at 120 °C.

Disulfide bonds (-S-S-) exhibit reversible cleavage and reformation under mild conditions, in our case facilitated by the presence of thiolate species formed during the epoxy/tiol-ene photopolymerization, as pointed out in the mechanism exhibited in Scheme 1. The self-healing process in disulfide-containing polymers follows a series of steps: when the polymer network undergoes mechanical stress or fracture, covalent disulfide bonds are disrupted, leading to cleavage at some points of the crosslinked network. The thiolate species initiates disulfide bond exchange, serving as reactive intermediates for bond reconstruction. When a thiolate encounters a disulfide bond, it attacks one of the sulfur atoms, leading to bond cleavage and the formation of a new disulfide linkage. In this way the broken disulfide linkages reorganize, restoring connectivity and reinforcing polymer integrity. The network structure is repaired, restoring mechanical properties without requiring external intervention. This process is described in Scheme 4:

The self-healing behavior also depends on the interplay between rigid and soft segments within the network. Upon heating, the flexible polythioether chains gain segmental mobility, while the rigid domains, originally vitrified at lower temperatures, soften near or above T_g_. This combined activation allows chain segments near the fractured interface to reorient, diffuse, and approach one another within the elastic window, effectively restoring network continuity. To assess the intrinsic healing capability of the polymers derived from the quaternary formulation, two specimens were evaluated: a reference test specimen and a healed counterpart, both derived from the same formulation and cured under identical conditions. The reference specimen was analyzed directly, while the healed sample was intentionally bisected and subjected to thermal healing for 20 h at 120 °C. The temperature-dependent storage modulus profile reveals that the healed specimen nearly reproduced the viscoelastic behavior of the original as shown in Figure 8. Specifically, the healed material achieved 94% recovery of the initial storage modulus across the temperature range examined. This substantial restoration suggests that the dynamic architecture of the crosslinked co-network allows effective reformation of crosslinks and retention of network integrity post-damage. Moreover, the healed curve closely parallels the reference profile, confirming that the healing process restored both stiffness and thermo-mechanical responsiveness. These results underscore the polymer’s potential for self-repair in high-performance applications, where maintaining mechanical function after damage is essential, particularly in additive manufacturing and functional coatings.

### 3.6. Determination of Mechanical Properties of the Prepared Polymer

**Figure 9:**
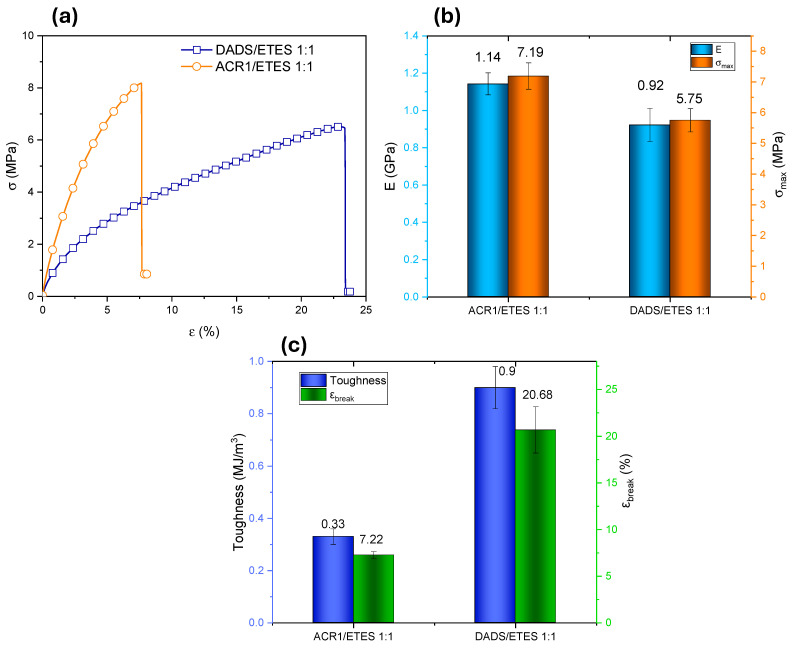
Summary of the tensile mechanical properties of the polymers derived from the quaternary formulations DADS:ETES 1:1 and ACR1:ETES 1:1: (**a**) stress (σ) vs strain (ε) curves; (**b**) elastic modulus (E) and maximum tensile strength (σ_max_); (**c**) toughness and ε_break_.

Due to the structural complexity of the synthesized poly(acrylate–co-ether–co-thioether) network, direct comparison with conventional polymers presents a challenge. To assess its tensile performance in a meaningful context, we conducted a comparative analysis against an analogous polymer system lacking disulfide bonds. For this purpose, trimethylolpropane triacrylate (ACR1), previously characterized by our research [17], was selected as a reference. ACR1 was chosen based on its high exothermicity during photopolymerization and the excellent mechanical properties of its resulting polymer network. Both formulations were prepared using an identical concentration of ETES to ensure consistency and isolate the effect of disulfide incorporation. As shown in Figure 9a, the DADS/ETES 1:1 system exhibits a linear elastic region up to 5% strain, followed by a strain-hardening phase prior to fracture, indicative of ductile behavior. In contrast, ACR1/ETES displays a shorter elastic region (up to 2% strain) and a sharp increase in stress leading to brittle failure. This difference suggests that the DADS-based network dissipates energy more effectively, likely due to its flexible aliphatic backbone and the dynamic nature of disulfide linkages. Figure 9b reveals that both formulations have comparable elastic modulus (E) and maximum tensile strength (σ_max), with ACR1/ETES showing slightly higher values (E = 1.14 GPa, σ_max = 7.19 MPa) than DADS/ETES (E = 0.92 GPa, σ_max = 5.75 MPa). The reduced stiffness in the DADS system could be attributed to a lower crosslink density, considering that ACR1 es trifunctional while DADS is bifunctional. However, the inclusion of disulfide bonds significantly enhances toughness and elongation at break: DADS/ETES achieves a toughness of 0.9 MJ/m^3^ and ε_break_ of 20.62%, representing a 186% increase over ACR1/ETES, as shown in Figure 9c. These findings highlight the trade-off between rigidity and resilience and underscore the mechanical advantages of incorporating dynamic covalent chemistry into polymer networks.

### 3.7. Three-Dimensional Printing

**Figure 10:**
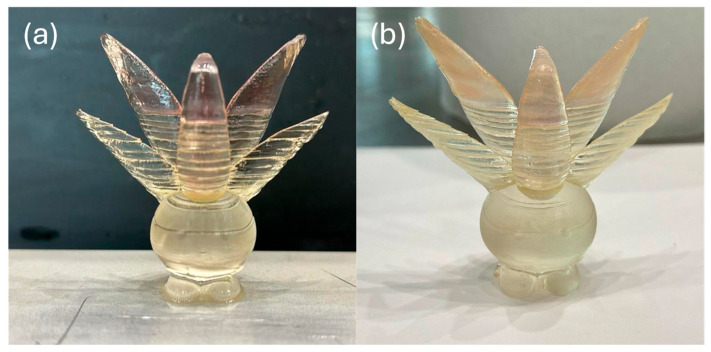
Three-Dimensional objects processed in a LCD printer using the quaternary formulation DADS:ETES 1:1, (**a**) after printing, (**b**) after dual post treatment, 1 h UV treatment at 40 mW/cm^2^ and 4 h at 120 °C.

The quaternary formulation (DADS:ETES) was successfully printed using a low-cost LCD-based 3D printer. To showcase the capabilities of the material, a geometrically complex object was selected, highlighting the formulation’s suitability for high-resolution additive manufacturing. As shown in Figure 10a, the printed object exhibited excellent dimensional fidelity and optical clarity immediately upon completion. To ensure full polymerization and enhance the mechanical integrity of the printed part, a dual post-curing protocol was applied: first, UV irradiation at an intensity of 40 mW/cm^2^ for 1 h in a controlled chamber, followed by thermal treatment at 120 °C for 4 h. Figure 10b displays the object after post-curing, where only a slight reduction in transparency was observed. This minimal change suggests that the formulation maintains its optical properties while achieving complete curing, making it well-suited for applications requiring both structural performance and visual clarity.

## 4. Research Outlook

This study demonstrates the potential of smart polymer networks fabricated via 3D printing. To expand their applicability, several improvements are proposed. First, enhancing sustainability and biocompatibility is essential. Given the toxicity of bisphenol A in EP, future formulations will explore biobased epoxy resins with aromatic structures to retain performance. Similarly, renewable multifunctional thiols may reduce environmental and health risks, making the materials more suitable for biomedical use.

Although components like pristine DADS and EP may pose cytotoxic risks in their uncured state, our dual post-curing protocol (UV followed by thermal treatment) ensures high conversion and minimizes residual monomers. This improves network stability and reduces leachables. Upcoming work will include cytotoxicity and extractables testing to confirm biomedical compatibility.

Self-healing conditions also warrant optimization. Current healing at 120 °C for 12 h can be improved. We propose incorporating catalysts such as tributyl phosphine, triethylamine, or imidazole to promote disulfide metathesis via thiolate formation. Adjusting catalyst levels and assessing their impact on curing and mechanical properties could enable healing under milder conditions. Additionally, photo-triggered systems may allow spatial and temporal control of healing, useful for soft robotics and biomedical devices.

From a manufacturing perspective, the high photoreactivity of the resin supports efficient photo-curing. While thermal post-treatment ensures full network formation, the dual-curing strategy balances speed and performance. To further streamline production, future efforts will explore optimized heating profiles, improved cure-through depth, and reduced oxygen inhibition. Adapting the formulation for high-throughput platforms like DLP or volumetric printing could accelerate fabrication of complex geometries without compromising material integrity.

Regarding durability, the material exhibits promising characteristics due to its hybrid architecture and post-curing strategy. The combination of acrylate, ether, and thioether linkages forms a densely crosslinked network with enhanced resistance to mechanical fatigue, thermal stress, and environmental degradation. Acrylate segments contribute rigidity and rapid curing, ether linkages provide flexibility and reduce brittleness, and polythioether domains—formed via disulfide exchange—introduce dynamic covalent character that supports self-healing and stress dissipation under cyclic loading. For applications involving prolonged exposure to moisture, heat, or mechanical cycling, further evaluation is essential. Future work will include accelerated aging tests, hydrolytic stability assessments, and fatigue analysis to rigorously quantify durability under realistic service conditions.

## 5. Conclusions

This work demonstrates the successful development of smart polymers with integrated shape memory and self-healing capabilities, fabricated via 3D printing using a quaternary photocurable formulation. The system comprises one equivalent of bis(2-methacryloyl)oxyethyl disulfide (DADS) and one equivalent of an epoxy/thiol-ene system (ETES), which includes diglycidyl ether of bisphenol A (EP), pentaerythritol tetrakis(3-mercaptopropionate) (PTMP), and the curing agent 4,4′-methylenebis(N,N-diallylaniline) (ACA4) in a 1:0.4:0.4 molar ratio. The resulting polymer forms a highly crosslinked poly(acrylate–co-ether–co-thioether) co-network, featuring both hard and soft segments that contribute to its shape memory behavior, while disulfide bonds embedded in the polyacrylate domains enable dynamic self-healing. The exothermic radical photopolymerization of DADS generates sufficient thermal energy to initiate curing of the ETES component at room temperature, allowing the formulation to be printed using a low-cost LCD 3D printer under ambient conditions. The printed polymer exhibited excellent shape memory performance, with shape fixity (R_f_) and shape recovery (R_r_) values of 93%. Self-healing was validated by bisecting test specimens and thermally treating them at 120 °C for 12 h, resulting in a 94% recovery of viscoelastic properties comparable to uncut controls. The smart polymers developed in this work open a wide range of potential applications across multiple sectors such as soft robotics and actuators, biomedical devices, and automotive and aerospace components, among others.

## Figures and Tables

**Table 1:** Components of the quaternary Acrylate/ETES formulation.

Component	Equivalents	Moles	Grams
DADS	1.00	3.4 × 10^−3^	1.00
EP	1.00	3.4 × 10^−3^	1.15
PTMP	0.40	1.34 × 10^−3^	0.66
ACA	0.40	1.34 × 10^−3^	0.48
BAPO	4 × 10^−3^	1.34 × 10^−5^	5.6 × 10^−3^

**Table 2:** Printing Parameters employed to obtain the designed object.

Printing Parameters	Base Layer	Rest of Layers
Resolution	70 μm	70 μm
Number of layers	30	228
Elevation after printing	5 mm	3 mm
Delay in turn off light	10 s	9 s
Exposition time	150 s	110 s
Overall Printing Time		9 h 7 min

**Table 3:** Shape Fixity (*R_f_*) and Shape Recovery (*R_r_*) values determined by DMA for the polyacrylate-polyether-polythioether interconnected hybrid co-network.

Cycle	$R_{f}$ (%)	$R_{r}\;(\%)$
1	94	93
2	93	93
3	93	93
4	93	92
Average	93	93

## Data Availability

Data is contained within the article.
